# *Saccharomyces boulardii* Strain CNCM I-745 Modifies the Mononuclear Phagocytes Response in the Small Intestine of Mice Following *Salmonella* Typhimurium Infection

**DOI:** 10.3389/fimmu.2019.00643

**Published:** 2019-04-02

**Authors:** Lidia Ibáñez, Rodolphe Pontier-Bres, Frederic Larbret, Akila Rekima, Valérie Verhasselt, Claudine Blin-Wakkach, Dorota Czerucka

**Affiliations:** ^1^CNRS, UMR 7370, LP2M, Faculté de Médecine, University Nice Sophia Antipolis, Nice, France; ^2^University Nice Sophia Antipolis, Nice, France; ^3^Ecosystems and Immunity team, Centre Scientifique de Monaco, Monaco, Monaco; ^4^EA6303, University Nice Sophia Antipolis, Hopital de l'Archet, Nice, France

**Keywords:** *Salmonella* Typhimurium, probiotic, *Saccharomyces boulardii* CNCM I-745, innate immunity, monocytes, *Lamina propria*, bone marrow, infection

## Abstract

Intestinal mononuclear phagocytes (MPs) comprise dendritic cells (DCs) and macrophages (Mφs) that play different roles in response to *Salmonella* infection. After phagocytosis, DCs expressing CD103 transport *Salmonella* from the intestinal tract to the mesenteric lymph nodes (MLN) and induce adaptive immune responses whereas resident Mφs expressing CX3CR1 capture bacteria in the lumen and reside in the *lamina propria* (LP) where they induce a local immune response. CX3CR1^+^ Mφs are generated from Ly6C^hi^ monocytes that enter the colonic mucosa and differentiate locally. We previously demonstrated that the probiotic yeast *Saccharomyces boulardii* CNCM I-745 (*S.b*) prevents infection by *Salmonella enterica* serovar Typhimurium (ST), decreases ST translocation to the peripheral organs and modifies the pro-and anti-inflammatory cytokine profiles in the gut. In the present study, we investigated the effect of *S.b* on the migratory CD103^+^ DCs and the resident CX3CR1^+^ Mφs. MPs were isolated from the LP of streptomycin-treated mice infected by ST with or without *S.b* treatment before or during the infection. In *S.b*-pretreated mice, we observed a decrease of the CD103^+^ DCs in the LP that was associated with the drop of ST recovery from MLN. Interestingly, *S.b* induced an infiltration of LP by classical Ly6C^hi^ monocytes, and *S.b* modified the monocyte-Mφ maturation process in ST-infected mice. Our results showed that *S.b* treatment induced the expansion of Ly6C^hi^ monocytes in the blood as well as in the bone marrow (BM) of mice, thus contributing to the Mφ replenishment in LP from blood monocytes. *In vitro* experiments conducted on BM cells confirmed that *S.b* induced the expansion of CX3CR1^+^ Mφs and concomitantly ST phagocytosis. Altogether, these data demonstrate that *Saccharomyces boulardii* CNCM I-745 modulates the innate immune response. Although here, we cannot explicitly delineate direct effects on ST from innate immunity, *S. b*-amplified innate immunity correlated with partial protection from ST infection. This study shows that *S.b* can induce the expansion of classical monocytes that are precursors of resident Mφs in the LP.

## Introduction

The intestinal innate immune system plays a crucial role in limiting microbial access to the gut tissue. Among the cells of the innate immune system, intestinal mononuclear phagocytes (MPs) comprise two distinct cellular populations that are essential for both the induction of active immunity and the maintenance of intestinal homeostasis. The first MP subclass, referred to as classical dendritic cells (DCs), is exclusively capable to migrate from the intestine to the mesenteric lymph nodes (MLNs) where they initiate adaptive immune responses [reviewed in (1)]. These DCs are routinely identified by their co-expression of αE integrin (CD103) and CCR7 that controls DC migration from the *lamina propria* (LP) to the MLNs (2–4). The second MP subclass, described in the literature as “non-conventional DCs” or “resident” macrophages (Mφs), resides in the intestinal LP (5, 6). These Mφs possess a high phagocytic activity and express CX3CR1, the fractalkine receptor (CX3CR1^+^ Mφs).

Depending of the organ, tissue-resident Mφs display different origin [reviewed in (7)]. In case of the gut, the yolk sac-derived macrophages are present in newborn mice but do not persist in the gut of adult mice. Bain et al. (8) demonstrated that the intestine of adult mice requires continuous replenishment of the LP macrophage pool from hematopoiesis-derived monocytes. This process occurs in young mice (2–3 weeks old) and coincides with the acquisition of commensal bacteria suggesting that microbiota regulates colonic Mφs pool in adult mice (8). This hypothesis was confirmed in germ-free mice that have lower levels of all monocyte and Mφ populations in the colon when compared to adult conventional mice. The monocyte-Mφ replenishment of the gut has been well documented during inflammation in mice and humans (9, 10). Initial studies using mice that express the diphteria toxin receptor (DTR) under the control of the CD11c promoter showed that after diphteria toxin-induced depletion of CD11c^+^ cells, Ly6C^hi^ monocytes enter the gut mucosa to replenish the Mφ compartment (11). These Ly6C^hi^ monocytes were originally named “inflammatory” given their tendency to enter inflamed tissues. Recent work shows that these monocytes can also enter non-inflamed tissues and are now referred as “classical monocytes.” In steady-state condition, these Ly6C^hi^ monocytes differentiate locally through short-lived CX3CR1^int^ intermediates and develop to mature F4/80^hi^MHCII^+^CX3CR1^hi^ resident Mφs that represent an anti-inflammatory cell population characterized by IL-10 expression and the capacity to induce regulatory T cells (Treg cells) (6). During colitis, the maturation of CX3CR1^int^ to anti-inflammatory CX3CR1^hi^ does not occur leading to the accumulation of pro-inflammatory CX3CR1^int^ cells in the LP (6). CX3CR1^hi^ macrophages also participate in antigen sampling by extending cellular extensions between epithelial cells to capture antigens or whole microbes and shuttle them across the mucosa to initiate immune responses in the LP (12, 13). In case of pathogenic infection by *Toxoplasma gondii* (14, 15), *Citrobacter rodencium* (16), and *Salmonella typhimurium* (17) classical Ly6C^hi^ monocytes and CX3CR1^int^ Mφs accumulate in large numbers in the intestinal mucosa of infected animals. In case of Salmonella sp. infection, after crossing the intestinal barrier, bacterial replication is controlled by neutrophils and monocytes that play a crucial role in host survival (18). *Salmonella* is transported to the MLNs via CD103^+^ DCs and infection becomes systemic when bacteria reach the blood (19). It has been recently demonstrated that intestinal challenge with *S. typhimurium* induces the migration of high phagocytic monocytic MPs (CX3CR1^hi^ Mφs) in the LP shortly after the infection (20). CX3CR1-deficient mice displayed an increased susceptibility to *Salmonella* infection suggesting that these cells are directly implicated in the host defense against pathogenic microorganisms (12). It was recently demonstrated that in the initial stage of infection by Salmonella, CX3CR1^+^ cells migrated to the intestinal lumen and internalized the bacteria limiting thereby the number of pathogens crossing the epithelium (21).

The yeast *Saccharomyces boulardii* CNCM I-745 (*S.b*) is used as probiotic for the prevention of bacterial infections (22). We previously depicted its mechanism of action in the context of Salmonella infection. Our results *in vitro* and *in vivo* demonstrated that *S.b* prevents ST infection and that the binding of ST to the *S.b* cell wall is part of the protective effects of the yeast (23). However, a recent study conducted *in vivo* clearly showed that *S.b* can modify the balance between pro- and anti-inflammatory cytokines in the LP (24). These results support the hypothesis that *S.b* acts as an immune-modulator that protects against ST infection in mice. Interactions between *S.b* and DCs were reported in mice after antibiotic treatment and in *in vitro* studies with DCs from patients with inflammatory bowel disease (25–27). Therefore, the aim of the present study was to evaluate the effect of *S.b* on MP (DC and Mφ) responses during ST infection. For this purpose, streptomycin-pretreated mice were infected with ST alone or in combination with *S.b*. We demonstrated that yeast treatment significantly decreased the CD103^+^ DC population in the LP of ST-infected mice as well as the number of viable bacteria recovered from the MLNs. *S.b* also induced an expansion of Ly6C^hi^ monocytes in the blood and BM. These cells infiltrated the LP and matured into Ly6C^low^ Mφs. A direct effect of *S.b* on the expansion of CX3CR1^int^ phagocytes was confirmed *in vitro*.

## Materials and Methods

### Bacterial and Yeast Strains

*Salmonella enterica* serovar Typhimurium SL1344 (ST) used in this study is a virulent streptomycin-resistant strain kindly provided by Stéphane Meresse, Center d'Immunologie de Marseille-Luminy, CNRS-INSERM Université de la Méditerranée, Marseille, France. ST were stored in Luria-Bertani (LB) medium supplemented by 15 % glycerol at −80°C and grown in LB broth overnight at 37°C without shaking. *S. boulardii* strain CNCM I-745 (*S.b*) (Ultra-Levure, BIOCODEX, France) cultures were obtained by inoculating a commercial lyophilized preparation of the yeast that was grown overnight at 37°C, with shaking, in Halvorston minimal medium (28).

### Mouse Experiments

Female, 6- to 8-weeks old C57BL/6 mice from specific pathogen-free stocks were purchased from Harlan (Harlan, France). During the course of these studies, sentinel animals were screened for common murine pathogens every 2 months. All animals were housed in individual HEPA-filtered cages with sterile bedding and had free access to sterilized water and food. All animal experiments were performed in accordance with the Animals Scientific Procedures Act (1986) and were approved by the Institutional Ethics Committee on Laboratory Animals (CIEPAL-Azur, Nice Sophia-Antipolis, France) (PEA Number: NCE/2013-68).

### Challenge of Mice With *S.b* and ST

*Salmonella enterica* serovar Typhimurium (ST) were grown in LB medium with streptomycin at 37°C without shaking until the late exponential phase. After centrifugation the bacterial pellet was washed twice with PBS and re-suspended in PBS. CFU were determined by plating serial dilutions of cultures on LB-agar medium containing streptomycin. Mice infected by ST do not develop intestinal inflammation and gastroenteritis but rather a systemic typhoid-like infection. This is due to the colonization resistance associated to microbiota. To overcome this resistance, we used an enteritis model described by Barthel et al. (29) with some modifications. Oral treatment with streptomycin reduced the microbiota by >80% and disrupted the colonization resistance during 2–3 days after antibiotic treatment (30). In brief, mice were deprived of water and food during 4 h before the administration of 20 μg of streptomycin/mouse by oral gavage. Two hours after antibiotic administration, food and water were provided *ad libidum*. Forty-eight hours after oral streptomycin treatment, water and food were once again withdrawn during 4 h, after which 200 μl of PBS containing ST (10^8^ CFU) were administered by oral gavage. Mice challenged with *S.b* received by gavage 200 μl of PBS containing ST (10^8^ CFU) and 10^7^ CFU of yeast at the same time and constituted the co-treated group (co-*S.b* group). In the case of the group pre-treated by *S.b* (pre-*S.b*), yeast was administered 2 days before infection together with streptomycin and again at the same time as infection. Two groups of control mice were used, one received 200 μl of PBS and the other received 200 μl of PBS containing 10^7^ CFU of yeast. The different groups are summarized in Figure S1. All the mice were sacrificed 3 days after infection.

### Cell Extraction From Small Intestinal Lamina Propria and Bone Marrow

After sacrifice, intestinal tissue was opened longitudinally and washed several time with PBS. After Peyers' patch (PP) excision, the LP was cut into 1 cm pieces, washed with RPMI 1,640 containing 50 μg/ml of streptomycin (Sigma) and incubated in RPMI 1,640 containing 3% FCS, 5 mM EDTA and 0,145 mg/ml DTT (Sigma) for 20 min at 37°C. The digested tissue was passed through a cell strainer (70 μm Nylon, BD). The tissue collected on the filter was then washed several times in RPMI containing 2 mM EDTA, and then digested in RPMI 1,640 containing 10 μg/ml liberase (Roche) and 100 μg/ml DNase (Roche) for 25 min at 37°C on a shaking platform. The digested tissue was once again passed through a cell strainer (40 μm Nylon, BD) and the collected cells were enumerated after trypan blue staining. These cells were then stained with a cocktail of antibodies. Cells were collected from BM by crushing the femora into small pieces and vigorous pipetting as previously described (31). Single cell suspensions were incubated with CD11b^+^ magnetic beads for monocytes enrichment, according to the manufacturer instruction (Miltenyi-Biotech).

### Analysis of ST Loads in MLNs

MLNs were removed and homogenized through a cell strainer (70 μm Nylon, BD) with the piston of a 10 ml syringe in Hank's Balanced Salt Solution (HBSS) at 4°C. Serial dilutions were plated on Luria Bertani-agar medium (LB-agar) containing 50 μg of streptomycin per ml for Colony Forming Units (CFU) determination.

### *In vitro* Experimentation

CD11b^+^-enriched monocytes from BM were cultured in αMEM medium containing 0.5% (v:v) of β-Mercapto-Ethanol and 5% (v:v) of Foetal bovin serum, supplemented or not with yeast for 24 h before infection. For the infection, *S. typhimurium* was added at the multiplicity of infection (MOI) of 10 bacteria/cells for 1 h. The cells were then washed and exposed to gentamycin at 100 μg/ml. After 1 h incubation, the medium was replaced by a medium containing 10 μg/ml of gentamycin and cells were incubated in this medium overnight. The next day, part of the cells was used for the invasion test by plating dilution on LB-agar containing 50 μg of streptomycin per ml for CFU determination, and the other part of the cells was used for phenotypic characterization by flow cytometry.

### Flow Cytometry

Cells were incubated on ice for 45 min in Fc block in the presence of relevant primary antibodies. The anti-CD11c FITC, anti-CD11b PE, anti-CD103 PECy5, anti-MHCII APC-efluor780, anti-F4/80 efluor450, anti-CD45 BV510, anti-CX3CR1 PECy7, anti-Sca1 PE, anti-CD16/32 PerCP, anti-CD34 efluor450, anti-B220 FITC, anti-Gr1 FITC, anti-Ter119 FITC, anti-CD3 FITC, and anti-CD127 APC were purchased from eBioscience. The anti-CD11b PE, anti-CD11c APC, anti-Ly6C FITC, anti-CD117 PECy7 and anti-CD43 PerCP were purchased from BD Bioscience. After staining, cells were analyzed by flow cytometry on a FACS Canto. Data were analyzed with the FlowJo software.

### Statistical Analysis

For all studies, data are expressed as means ± SEM and groups were compared using non parametric tests:Kruskal-Wallist/Dunn's test or Mann-Whitney test. All analysis were performed using GraphPad Prism Sofware with statistical significance accepted for *p* < 0.05.

## Results

### *S.b* Decreases the Recovery of ST From MLNs and the Proportion of CD103^+^ DCs

**Figure 1:**
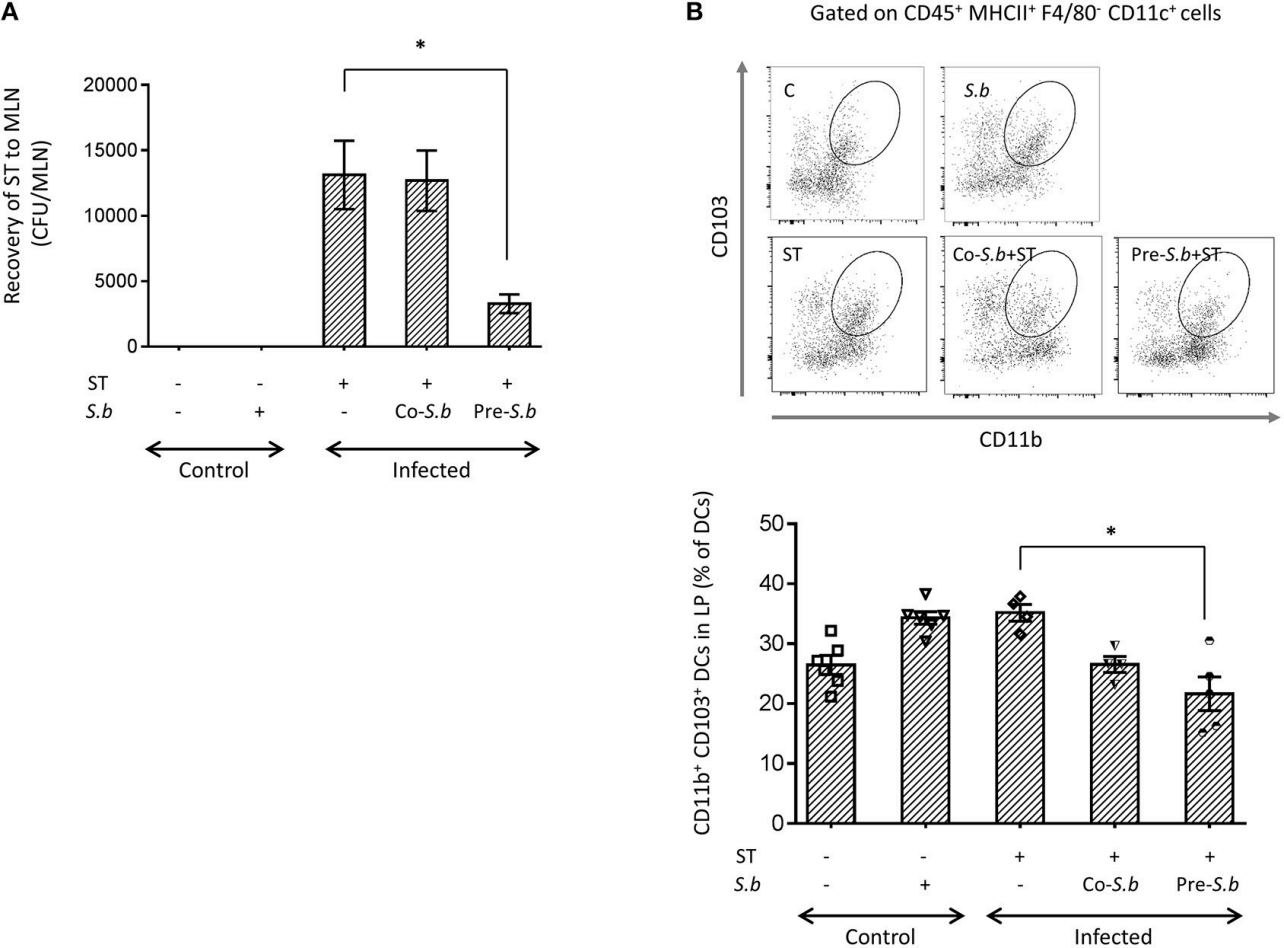
*S.b* treatment reduces ST translocation to the MLNs and CD103^+^ DCs in the LP of Salmonella-infected mice. Mice were infected intra-gastrically for 3 days and LP and MLNs were dissected for further analyzes. **(A)** The number of viable ST bacteria recovered from the MLNs was determined in control mice (treated or not by *S.b*) and in mice infected with ST alone or infected in the presence of *S.b* (co-*S.b*) or pretreated with the yeast before infection (pre-*S.b*). MLNs were dissociated and aliquot plated on LB-agar streptomycin for counting CFUs. **(B)** Flow cytometry plots of the CD103^+^ DC subset in the LP. After monocytes extraction from LP, single cell suspensions were prepared and stained with anti-CD45, -CD11b, -F4/80, -CD11c, -MCHII, and -CD103. CD45^+^ cells were further gated according to F4/80 and MHC-II expression and the DC population was defined as MHC-II^+^F4/80^−^ cells. These cells were gated according to the expression of CD11c and CD11b. The CD11c^+^CD11b^+^ population was gated for CD103. Histogram of CD11b^+^CD103^+^ DCs in control mice (treated or not by *S.b*) and mice infected with ST alone or infected in the presence of *S.b* (co-*S.b*) or pretreated with the yeast before infection (pre-*S.b*). Each symbol represents an individual mouse: small horizontal lines indicate the mean (± SEM). The data are combined from two independent experiments. ^*^p < 0.05.

Stimulation of the innate immune response is an essential mechanism of the host to prevent infection. We previously demonstrated that *S.b* could modify the cytokine expression pattern in the early phase of infection by ST, which was beneficial for the host survival and prevented the loss of body weight during the late phase of infection (24). This beneficial effect was also correlated with a decrease of bacterial translocation from the gut to other organs. We, and others have previously shown that the Salmonella number in the intestine was not affected by *S. b* treatment despite a beneficial effect (24, 32). We thus investigated the effect of *S.b* on the migratory phagocytes in the LP. To determine the effect of *S.b* on ST spread, mice were either pre-treated by the yeast before infection (pre-*S.b)* or co-treated by the yeast (co-*S.b*) at the time of infection by ST. Three days post-infection, the bacterial burden was assessed in the MLNs. The bacterial burden was not impacted when *S.b* was given at the time of infection (co-*S.b*) but was significantly decreased in mice from the pre-*S.b* group (Figure 1A). As CD103^+^ DCs constitute the migratory cells that translocate ST from the LP to the MLNs (19), this result suggests a lower traffic of these cells from the LP to the MLN of treated mice. Unexpectedly, analysis of this population revealed that it was decreased in the LP by *S.b* treatment (Figure 1B). Alternatively, this result may reflect a switch in LP's monocytic cell population toward another monocytic population such as Mφs. We thus investigate the effect of the yeast on other myeloid population in the LP.

### *S.b* Induces the Maturation of Intestinal Ly6C^hi^ Monocytes Into Ly6C^−^ Mφs

**Figure 2:**
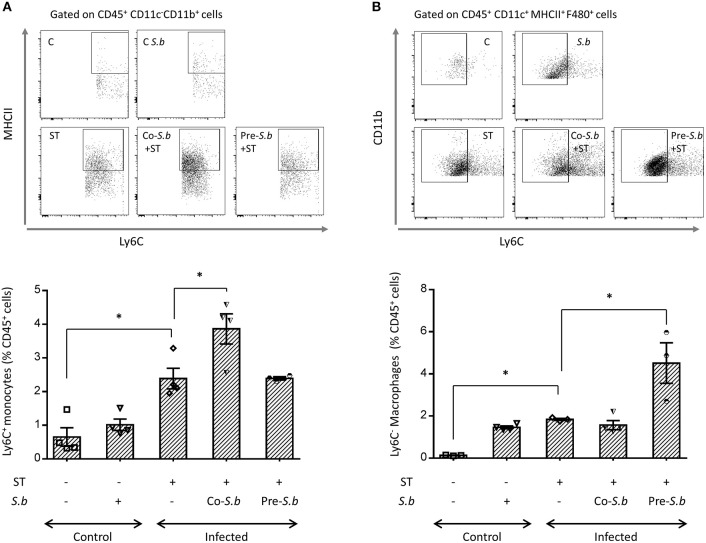
*S.b* modified the maturation of Ly6C+ monocytes into resident Ly6C - Mφs. Mice were infected intra-gastrically for 3 days and the LP was dissected. Flow cytometry plots of Ly6C macrophage subset in the LP. After monocyte extraction from the LP, single cell suspensions were prepared and stained with anti-CD45, -CD11b, -F4/80, -CD11c, -MHC-II, and -Ly6C. **(A)** For the Ly6C^hi^ monocytes, the different populations of the cascade of maturation were identified by the expression of MHC-II and Ly6C among CD11c^−^CD11b^+^ monocytes. **(B)** The macrophage populations of the cascade were identified by the expression of Ly6C and CD11b among CD11c^+^MCH-II^+^F4/80^+^ macrophages. Histograms representing the population of Ly6C^hi^ monocytes in **(A)** and Ly6C^neg^ macrophages in **(B)** in control mice (treated or not by *S.b*) and mice infected with ST alone or infected in the presence of *S.b* (co-*S.b*) or pretreated with the yeast before infection (pre-*S.b*). The gating strategy is summarized in Figure S2. Each symbol represents an individual mouse: small horizontal lines indicate the mean (± SEM). The data are combined from two independent experiments. ^*^p < 0.05.

Crossing the epithelial barrier is thought to be the initial step of ST infection. In the intestine, a subset of Mφs has been shown to sample Salmonella (12). This population rises from Ly6C^hi^ monocytes that are recruited into the mucosa and differentiate locally toward Ly6C^−^ Mφs. This process occurs through short-lived intermediates with down-regulation of Ly6C, and up-regulation of MHC-II and F4/80 (6). In the LP of ST-infected mice, we observed an increase of Ly6C^hi^ monocytes that are known to be at the origin of the maturation process toward Mφs (Figure 2A). This maturation process has been confirmed by the augmentation of resident Mφs that had lost the phenotypic marker Ly6C (Figure 2B). The kinetics of the monocyte-to- Mφ maturation process induced by ST was modified by *S.b* treatment (Figures 2A,B). We observed an increase in the intermediate population expressing MHC-II and Ly6C (Figure 2A) in mice co-treated with the yeast during infection (co-*S.b*) corresponding to 3 days of treatment by *S.b*. In the pre-*S.b* group that received yeast for 5 days, the intermediate population was not modified (Figure 2A) whereas the LP content in mature resident Ly6C^−^ Mφs was significantly up-regulated when compared to ST infected mice or co-*S.b* treated group (Figure 2B). Three days of yeast treatment induced an increase of monocytes (Figure 2A) and 5 days of treatment increased the monocyte-derived Mφs pool in the LP (Figure 2B). These findings suggested that *S.b* exerts a time-regulated turnover of the monocyte-to-Mφs maturation process in the LP. This hypothesis was confirmed in control mice treated by *S.b* (without infection) that displayed a higher proportion of both the Ly6C^hi^ monocytes as well as the resident Ly6C^−^ Mφs (Figures 2A,B). Altogether, these observations showed that *S.b* affects the monocyte-Mφ homeostasis and that this effect is dependent on the duration of yeast administration.

### *S.b* Increases Ly6C^hi^CX3CR1^+^ Monocytes in the Blood and BM

**Figure 3:**
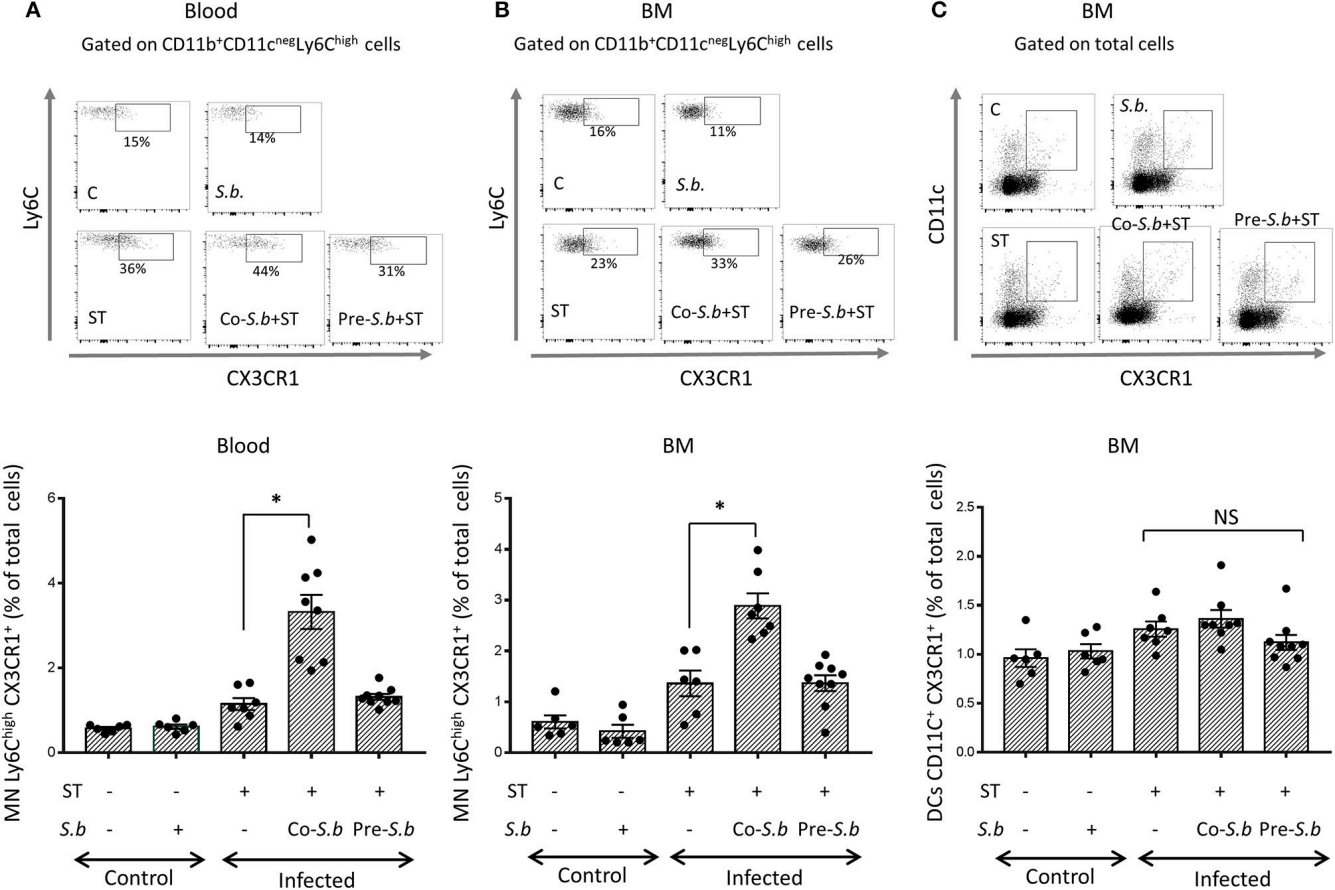
*S. b* up-regulates Ly6C^hi^ CX3CR1^+^ monocytes in the blood and in the BM but has no effect on CX3CR1^+^ DCs. **(A)** Flow cytometry plots of Ly6C monocyte subset in the blood. After immune cell extraction from the blood, single cell suspensions were stained with anti -CD11b, -CD11c, -Ly6C, and CX3CR1. Gating of Ly6C^hi^ CX3CR1 monocytes from CD11b+ CD11c^neg^ subsets. **(B)** Flow cytometry plots of BM Ly6C^hi^ monocytes. Single cell suspension was stained with anti -CD11b, -CD11c, -Ly6C, and CX3CR1. Gating of Ly6C^hi^ CX3CR1 monocytes among CD11b^+^ CD11c^neg^ subsets. **(C)** Gating on CX3CR1^+^ cells among CD11c^+^ DC population. Histograms represent the different populations from control mice (treated or not by *S.b*) and mice infected with ST alone or infected in the presence of *S.b* (co-*S.b*) or pretreated with the yeast before infection (pre-*S.b*). Each symbol represents an individual mouse: small horizontal lines indicate the mean (± SEM). The data are combined from two independent experiments.

Bain et al. (8) demonstrated that in adult mice, intestinal macrophages are derived from conventional hematopoiesis. Thus, we investigated the presence of Ly6C^hi^CX3CR1^int^ monocytes in the blood and BM of mice infected or not by ST. Infection by ST had a tendency to increase the percentage of Ly6C^hi^ CX3CR1^int^ monocytes in the blood and the BM when compared to control mice (Figures 3A,B). In the blood of co-*S.b* mice, the proportion of monocytes expressing Ly6C^hi^ and CX3CR1^int^ was higher when compared to ST-infected animals. The co-stimulatory effect of the yeast on classical monocytes disappeared when the yeast was administered prior to the infection (pre-*S.b* group) (Figures 3A,B). Thus, similarly to what we observed in the LP (Figure 2A), we observed in the blood and the BM a time-scale stimulatory effect of *S.b* on the population of monocytes that are known to give rise to mature Mφs in the LP. Three days of treatment by *S.b* increased classical Ly6C^hi^ monocytes in the blood and BM of infected mice whereas no effect was observed after 5 days of treatment (Figure 3A).

The Ly6C^high^CX3CR1^+^ monocytes in the blood are recruited from the BM. We thus quantified this population in the BM of infected or non-infected mice (Figure 3B). This population increased in ST infected mice and in the co-*S.b* group, but not in the pre-*S.b* group, as observed in the blood. Contrasting with Ly6C^high^CX3CR1^+^ monocytes, the CX3CR1^+^ DC population in the BM was not affected by *S.b* treatment (Figure 3C).

**Figure 4:**
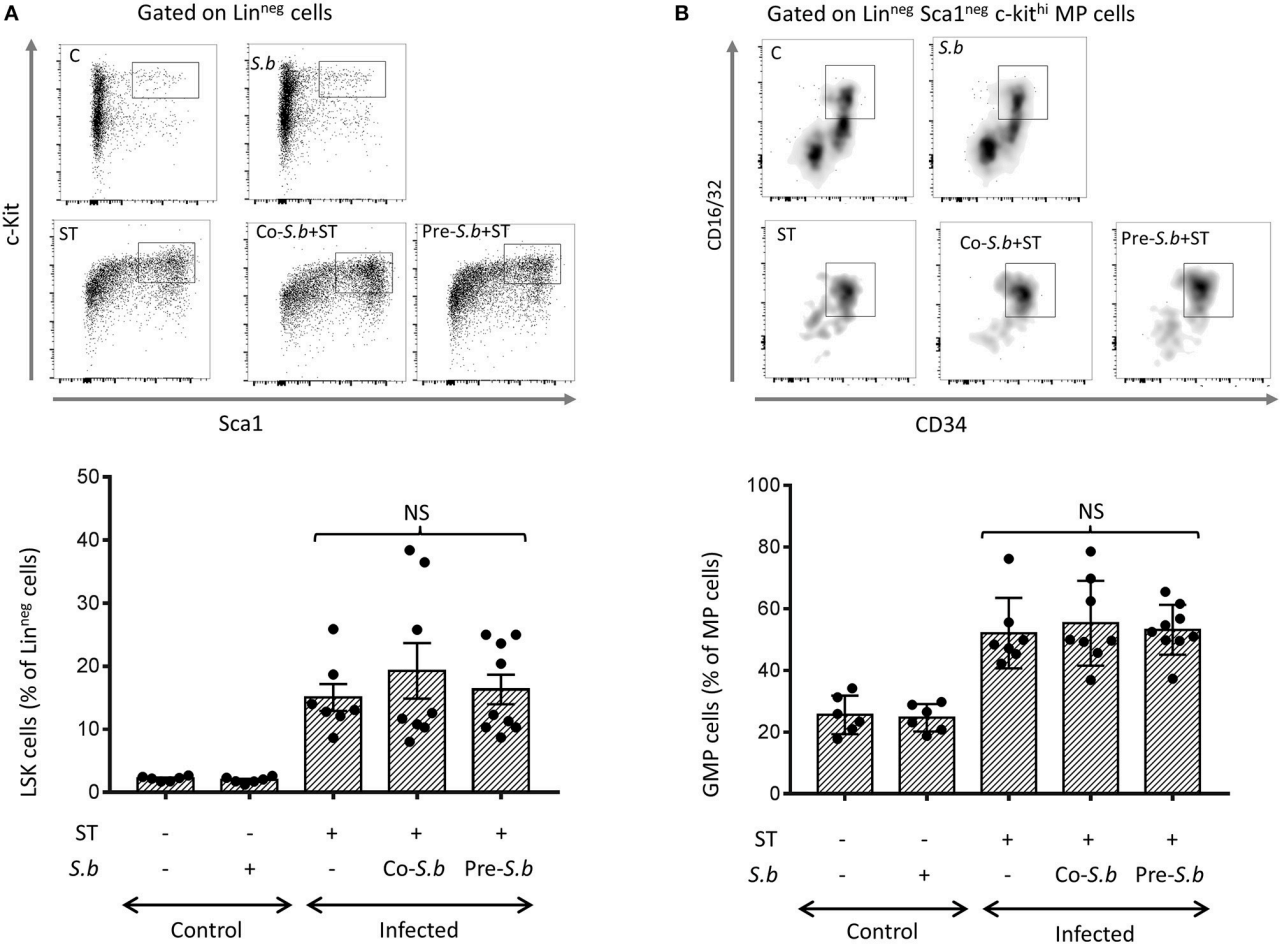
*S.b* did not modified hematopoiesis in BM of infected mice. **(A)** Flow cytometry plots of LSK cells among Lin^neg^ cells. Cells were stained with anti-Sca1 and anti-Kit antibodies. **(B)** Flow cytometry plots of GMPs among Lin^neg^ Sca ^neg^ c-Kit MP cells after staining with anti-C16/32 and ant-CD34 antibodies. Histograms represent the different populations from control mice (treated or not by *S.b*) and mice infected with ST alone or infected in the presence of *S.b* (co-*S.b*) or pretreated with the yeast before infection (pre-*S.b*). Each symbol represents an individual mouse: small horizontal lines indicate the mean (± SEM). The data are combined from two independent experiments.

As the CX3CR1^+^ Mφ progenitors were up-regulated in infected-mice treated with *S.b* for 3 days (Figures 3A,B), we investigated the effect of *S.b* on early hematopoietic progenitors generated in the BM. Monocytes were constantly generated in the BM from hematopoietic stem cells (HSCs) via granulocyte-macrophage progenitors (GMPs). As expected, Lin^−^ Sca1^+^ c-kit^+^ (LSK) progenitors that contain HSCs, and GMP progenitors are increased in ST infected-mice when compared to control mice but were not affected by *S.b* treatments (Figures 4A,B). We concluded that the up-regulation of the Ly6C^hi^CX3CR1^+^ monocytes observed in mice treated by the yeast was not associated to an increase of their progenitors.

### *S.b* Induces the Expansion of BM Ly6C^hi^ and CX3R1^int^ Monocytes

**Figure 5:**
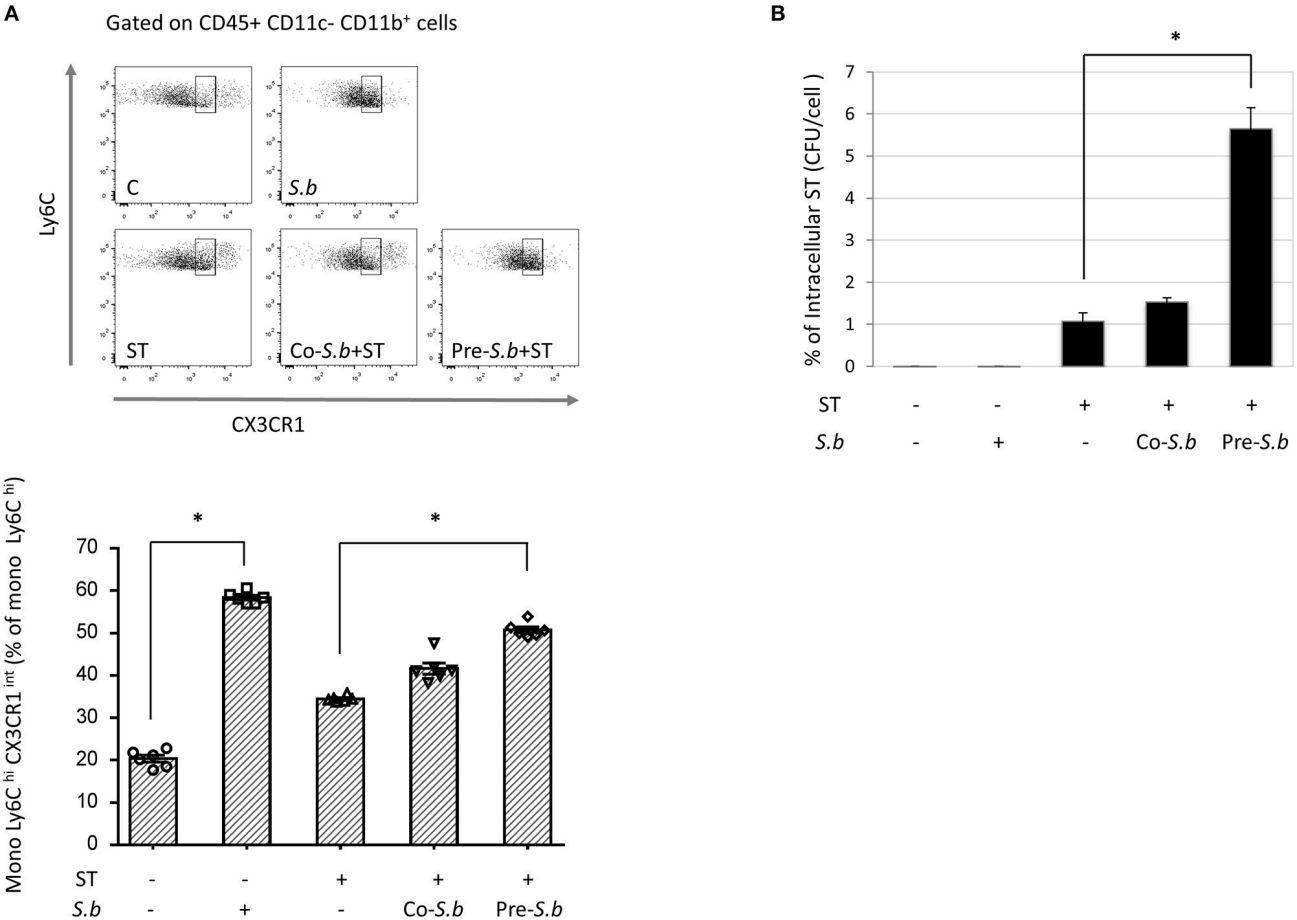
*S.b* induces the expansion of monocytes Ly6C^hi^ and CX3CR1^+^ BM monocytes and the phagocytosis of ST. Freshly extracted BM monocytes were enriched by anti-CD11b^+^ beads treatment. Cells were exposed or not to *S.b* overnight and infected by ST for another 24 h with gentamycin treatment to kill extracellular bacteria (for details see Materials and Methods). **(A)** Flow cytometry plots of Ly6C^hi^ CX3CR1^int^ monocytes were performed on CD45^+^ cells after identification of the CD11b^−^CD11c^+^ population. The gating strategy is summarized in Figure S3. **(B)** Determination of intracellular bacteria number by plating on agar and counting of CFUs. Conditions: control (treated or not by *S.b*), infected by ST alone or infected in the presence of *S.b* (Co-S.b) or exposed overnight to *S.b* before infection (Pre-*S.b*). The experiments were performed 3 times. ^*^p < 0.05.

The results reported above suggest that *S.b* might drive the expansion of monocytes. To explore this hypothesis, we performed an *in vitro* experiment on BM cells infected or not with ST. Flow cytometry analysis clearly showed that overnight incubation with *S.b* highly increased the proportion of Ly6C^hi^CX3CR1^int^ monocytes (Figure 5A). Infection by ST also induced this population but in a lower proportion than the yeast. In cells exposed to yeast and then infected (pre-*Sb*), the proportion of Ly6C^hi^CX3CR1^int^ monocytes was significantly increased when compared to ST-infected cells. The number of intracellular bacteria found in the different conditions was determined by the Gentamycin protection assay (Figure 5B). In yeast-pretreated cells, we observed a significant increase of intracellular bacteria when compared to ST-alone infected cells confirming the high phagocytic property of these cells after yeast treatment. These findings clearly showed that *S.b* induced *in vitro* the expansion of highly phagocytic Ly6C^hi^CX3CR1^int^ monocytes.

## Discussion

It is now well established that the intestinal immune response constitutes a highly dynamic process that controls exchanges between the lumen and the mucosa. In steady-state condition, this process controls tolerance against alimentary antigens and commensal microbiota while, in pathological conditions, it controls harmful microbe exclusion and inflammatory responses. Two populations of MPs were identified in the LP as major players in pathogen exclusion: DCs and Mφs. Only DCs expressing CD103 and CCR7 are able to migrate from the LP to the mesenteric lymph nodes and initiate the adaptive immune responses by priming naïve T cells. By contrast, Mφs expressing the fractalkine receptor CX3CR1 are resident in the LP where they act as effector cells of innate immunity. They engulf and clear bacteria, produce cytokines and maintain intestinal homeostasis. In this study, we showed that administration of *Saccharomyces boulardii* strain CNCM-I745 to mice infected by *Salmonella typhimurium* alters the distribution of DCs and Mφs in the LP. Yeast administration to mice before infection decreases the pool of CD103^+^ DCs and increases the monocyte-Mφ pool in the LP. These effects are associated *in vivo* with the expansion of Ly6C^hi^ CX3CR1^int^ monocytes in the blood and BM in *S.b*-treated mice before infection. Data presented in this study also show that *S.b* administration alone modifies the myeloid phenotype of monocytes in the LP in streptomycin-treated mice. A direct effect of the yeast on myeloid differentiation (or maturation) phenotype was confirmed *in vitro* on BM-derived monocytes exposed to the yeast. Altogether, our results demonstrate that *S.b* modifies the myeloid phenotype in the intestine that could account for the prevention of infection.

The anti-infectious properties of *S.b* are well documented in clinical reports but its mechanisms of action against pathogens are partially understood (22, 33, 34). Most experimental research from *in vitro* studies on cell cultures and animal models has been focused on the prevention of microbial pathogen adherence, translocation of the commensal microbial flora, neutralization of bacterial toxins (i.e., *Clostridium difficile* toxin A or cholera toxin), toxin-related signaling, maintenance of normal intestinal permeability and barrier function, as well as control of epithelial electrolyte transport and luminal secretion (35). The concept that probiotics can interact with immune cells constitutes the foundation for the immuno-regulatory effect of these microorganisms. For *S.b*, such interactions with DCs were reported in the context of ulcerative colitis (UC) and Crohn's diseases (CD) (26, 27). The authors investigated the effect of *S.b* supernatant on the stimulation by LPS of DCs isolated from healthy volunteers or patients with UC or CD. As incubation with fungal supernatant decreases the expression of the DC co-stimulatory molecules CD40, CD80 and of CCR7, the authors concluded that, in case of chronic inflammation, *S.b* exhibits in part its anti-inflammatory potential through modulation of the DC phenotype, migration and T polarization capacity. In the present study, we have investigated the effect of *S.b* on MPs in the case of acute inflammation occurring during ST infection. Experimentations were conducted in streptomycin-pretreated mice. This model enforces pathogen invasion through the LP rather than PPs and provides a clinically relevant mouse model of Salmonellosis (29). In this model, the CD103^+^ DCs were demonstrated to be the first DCs that carried intracellular Salmonella after oral infection (19). Our data show that when *S.b* is administrated before infection, the intestinal population of CD103^+^ DCs is decreased when compared to ST-infected animal. This can be explained by the migration of this population away from the LP and consequently by an increased trafficking to MLN. However, in these mice the bacterial burden is reduced in the MLNs. One hypothesis to explain this is that the CD103^+^ DCs that migrate to the MLN are translocating dead bacteria after *S.b* treatment that cannot be detected by our CFU analysis. The second hypothesis is that *S.b* modified the myeloid phenotype of MPs resulting in the decrease of the CD103^+^ DC population that can benefit to the rise of other pool of myeloid cells.

In the streptomycin mouse model, *S. typhimurium* uses at least two mechanisms to cross the epithelial barrier. The first one known as the “classical pathway” involves bacterial interaction with the epithelial cells. The second one named “alternative pathway” occurs after ST translocation through the epithelium and involves interaction between bacteria and innate immune cells (36). Among them, the CX3CR1 compartment represents a monocyte-to-Mφ differentiation continuum, ranging from Ly6C^hi^CX3CR1^int^ monocytes at one end to mature CX3CR1^hi^ Mφs at the other. This process involves down-regulation of Ly6C, and up-regulation of MHC-II and F4/80 (6). Our data showed that ST infection induces the recruitment of Ly6C^hi^ monocytes to the LP in the intestine. Concomitantly, we observed an increase of Ly6C^−^ Mφs expressing MHC-II and F4/80. In mice treated by *S.b*, the recruitment of Ly6C^hi^ monocytes and their maturation process toward Ly6C^−^ Mφs are modified when compared to ST-infected animals. This effect is a time-scale event since we observed an important recruitment of Ly6C^+^ monocytes into the LP in mice exposed to the yeast for 3 days (co-*S.b* group). However, this effect was decreased in mice exposed for 5 days to the yeast (pre-*S.b* group). By contrast, we observed an important up-regulation of Ly6C^−^ Mφs in the LP of pre-*S.b* mice. These data strongly suggest that the yeast modulates the kinetics of monocyte-Mφ maturation. This hypothesis was confirmed in control mice treated only by the yeast. These mice display an increase of both Ly6C^hi^ monocytes and Ly6C^−^ Mφs when compared to control mice that did not receive the yeast. These data clearly demonstrate that *in vivo S.b* can modify myeloid cell phenotype in the LP.

The traditional view of the mononuclear phagocyte system is that the precursors develop in the bone marrow (BM), with mature monocytes then entering the circulation and migrating into the tissues to replenish tissue macrophages (37). There are two principle subsets of circulating monocytes: classical Ly6C^hi^ monocytes and non-classical Ly6C^low^ monocytes [reviewed in (38)]. Classical monocytes patrol in the tissues and pick up antigens to transport them to the draining lymph nodes. In the context of inflammation, these monocytes differentiate into macrophages [reviewed in (39)]. In the present study, we showed that ST infection induces classical Ly6C^hi^ CX3CR1^int^ monocytes in the blood and BM concomitantly to the recruitment of Ly6C^hi^ monocytes in the LP. Classical monocyte populations are up regulated in the blood and BM of mice co-treated by *S.b*. However, as shown in this study, *S.b* administration has no effect on the hematopoietic progenitors generated in the BM. Interestingly, the *in vitro* data obtained on BM monocytes clearly demonstrated that *S.b* induces the expansion of Ly6C^hi^ CX3CR1^int^ monocytes. This up-regulation was also observed in ST-infected cells that were treated by *S.b* before infection and was correlated with an increased number of intracellular bacteria. High phagocytic activity is an important feature of this immune cell population. Thus, we hypothesize that *in vivo S.b* also induces the expansion of classical monocytes in the BM that migrate through the blood to the LP.

Recruitment of Ly6C^hi^ monocytes from the BM into the circulation was reported in the case of infection by *L monocytogenesis* and these cells were involved in the defense against infection (40). This defense is a multistep process that involves CCR2-mediated emigration of Ly6C^hi^ monocytes from the BM to the bloodstream and CCR2-mediated recruitment of Ly6C^hi^ monocytes from the bloodstream to the infected tissues (41). CCR2-mediated monocyte recruitment is also essential for the defense against *Toxoplasma gondii, Mycobacterium tuberculosis* and *Cryptococccus neoformans* infection, revealing that inflammatory monocytes are also involved in the defense against protozoan and fungal pathogens [reviewed in (42)]. Thus, the expansion of Ly6C^hi^ CX3CR1^int^ monocytes in the BM and blood and the increased infiltration of classical monocytes in the LP in mice treated by *S.b* contribute to the protective effect of *S.b*. against ST infection.

Intestinal Ly6C^neg^ Mφs expressing CX3CR1 represent a cell population that plays a variety of roles. They maintain intestinal immune homeostasis in steady-state by controlling antigen access and, in case of infection, they are considered as part of the defense strategies against infection complementary to mucus protection and IgA secretion (43). During infection with *Toxoplasma gondii* (14, 15), *Citrobacter rodentium* (16) or *Salmonella typhimurium* (12, 21), Ly6C^hi^ monocytes and their descendants are the most efficient captors of pathogens and are indispensable for their elimination. The CX3CR1^hi^ subclass appears to be directly involved in sampling and uptake of luminal antigens and microbes by extending cellular processes between epithelial cells and shuttling them across the epithelial barrier (12, 13, 35). Thus, the acceleration of the Ly6C^hi^ monocytes turnover toward Ly6C^neg^ Mφs in the LP of mice treated by *S.b* contributes to the protection in case of Salmonella infection.

Bistoni et al. (44) reported in a study published in 1986 that administration of a non-virulent strain of yeast induces a protective effect against a second infection by virulent yeast but also bacteria that is mediated by granulocytes and macrophages lineage. In another study performed on human DCs, the authors have demonstrated that this effect is mediated by a Dectin-1 receptor that is able to recognize β-1,3 glycans which make up to 50% of the fungal cell wall (45). In our study, the yeast and bacteria were administered by gavage implying a direct contact of these microorganisms with the gut epithelium. In this context, we do not exclude a direct interaction of a compound of *S.b* cell wall with the myeloid cells in the LP. The second hypothesis is that *S.b* could secrete a factor or induce the secretion of factors by LP that modify the environment of immune innate cells and direct they maturation process. In the case of intestinal bowel disease, the supernatant from *S.b* culture modified DC activation status by LPS suggesting that the yeast can act trough a secreted factor (26). The mechanism of action of *S.b* in control non-infected mice as well in ST-infection is under investigation.

In summary, the present study shows that *S.b* may exhibit its anti-infectious potential through the modulation of the innate immune responses in the LP. This study opens new perspectives for the use of *S.b* as immunotherapy in infectious diseases.

## Author Contributions

LI, RP-B, and FL carried out the experimentation and analyzed the data. AR carried out the experimentation. LI and CB-W analyzed the data and participated in the writing of the first version of the manuscript. CB-W and VV provided support in experimental methods and help in data interpretation. DC conceptualized the project, designed experiments, analyzed the data, wrote the manuscript, edited the manuscript, and revised the final version. All the authors contributed to an enriching discussion. All authors read and approved the final manuscript.

### Conflict of Interest Statement

DC is a scientific advisor to Biocodex SA (Gentilly, France) and has received travel grant from them. DC and RP-B salaries are founded by BIOCODEX S.A. LI received a post-doctoral fellowship from Biocodex SA. CB-W received a grant from Biocodex SA. There are no patents, products in development or marketed products to declare. The remaining authors declare that the research was conducted in the absence of any commercial or financial relationships that could be construed as a potential conflict of interest.

## Supplementary Material

Figure S1 Mice treatment. Mice were subdivided in 5 groups: control group, S.b group, ST group, Co-*S.b*+ST group, and Pre-*S.b*+ST group. The experimentation started at day−2 with the orally administration of 20 μg of streptomycin (str) in 200 μl of PBS to all mice. Moreover, pre-*S.b*+ST group was treated by *S.b* (10^7^CFU in 200 μl of PBS). At day 0, control group was treated with PBS, *S.b* group was treated by *S.b* (10^7^CFU in 200 μl of PBS), ST group was treated by ST (10^8^CFU in 200 μl of PBS) and Co-*S.b*+ST and Pre-*S.b*+ST groups were treated at the same time by ST and *S.b* (10^8^CFU and 10^7^ CFU in 200 μl of PBS, respectively). At day 3 of experimentation, mice were sacrificed and the intestine, bone marrow and MLNs were removed and proceeded for cell isolation.

Figure S2 Gating strategy for the Ly6C ^hi^ monocyte and Ly6C^−^ macrophage identification in the LP.

Figure S3 Gating strategy for the identification of Ly6C^hi^CX3CR1^int^ population among BM cells.
