# Supplementary figures and images for: Saccharomyces boulardii Strain CNCM I-745 Modifies the Mononuclear Phagocytes Response in the Small Intestine of Mice Following Salmonella Typhimurium Infection
Source: Front Immunol. 2019 Apr 2;10:643. doi: 10.3389/fimmu.2019.00643 (PMC6455222; doi:10.3389/fimmu.2019.00643)

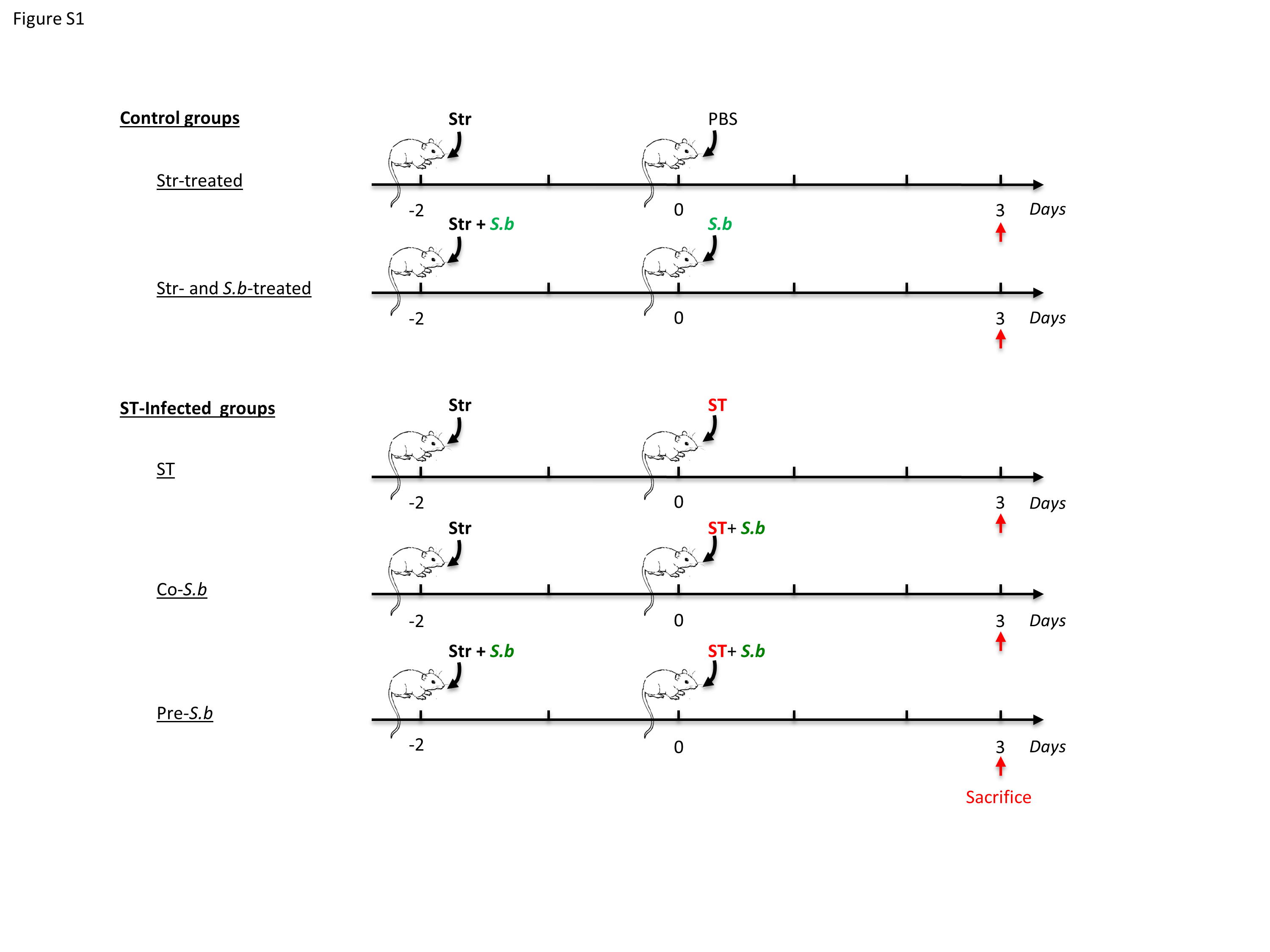

Supplement: Figure S1 — Mice treatment. Mice were subdivided in 5 groups: control group, S.b group, ST group, Co-S.b+ST group, and Pre-S.b+ST group. The experimentation started at day−2 with the orally administration of 20 μg of streptomycin (str) in 200 μl of PBS to all mice. Moreover, pre-S.b+ST group was treated by S.b (107CFU in 200 μl of PBS). At day 0, control group was treated with PBS, S.b group was treated by S.b (107CFU in 200 μl of PBS), ST group was treated by ST (108CFU in 200 μl of PBS) and Co-S.b+ST and Pre-S.b+ST groups were treated at the same time by ST and S.b (108CFU and 107 CFU in 200 μl of PBS, respectively). At day 3 of experimentation, mice were sacrificed and the intestine, bone marrow and MLNs were removed and proceeded for cell isolation. [file Image_1.tif]

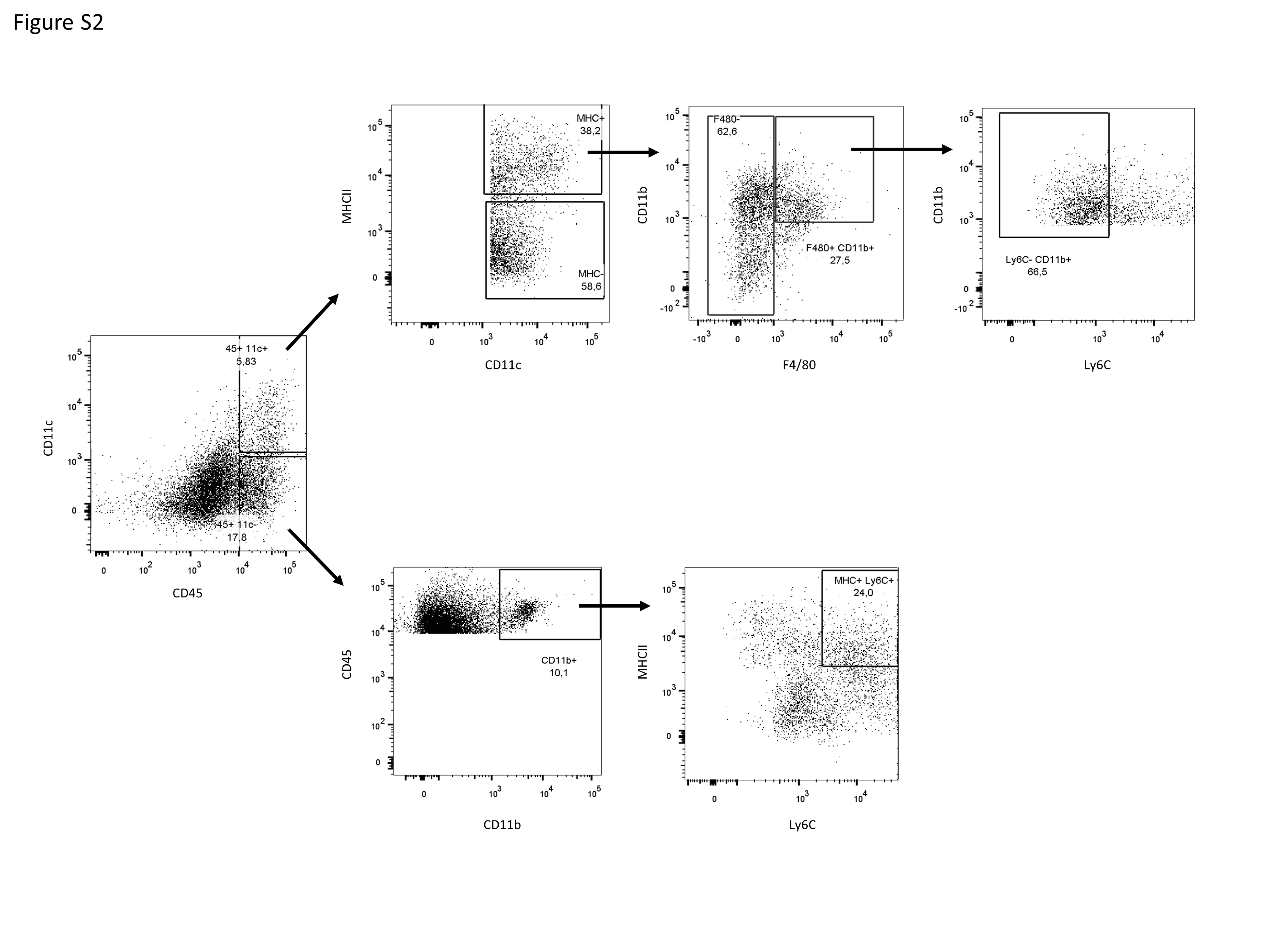

Supplement: Figure S2 — Gating strategy for the Ly6C hi monocyte and Ly6C− macrophage identification in the LP. [file Image_2.tif]

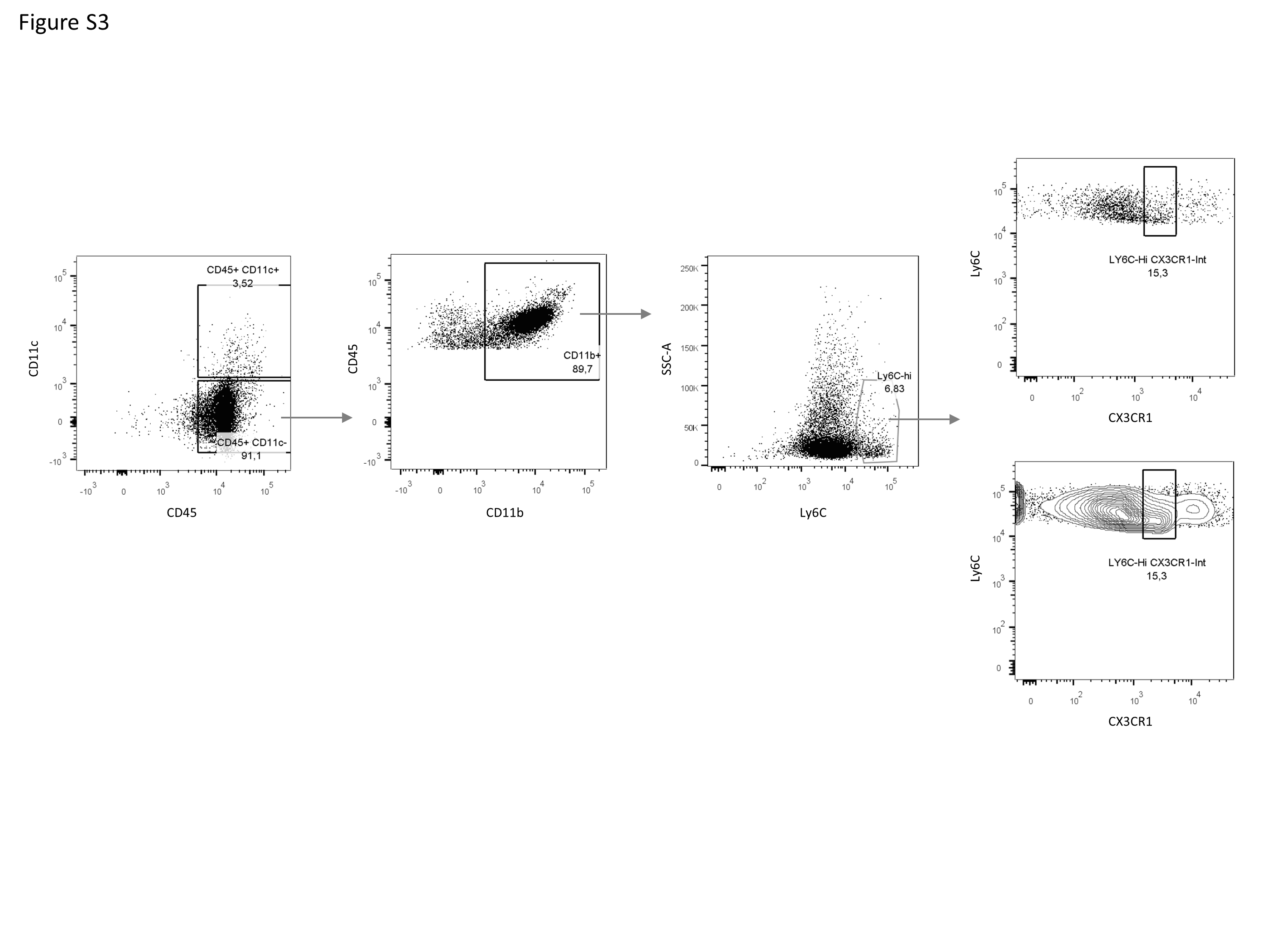

Supplement: Figure S3 — Gating strategy for the identification of Ly6ChiCX3CR1int population among BM cells. [file Image_3.tif]
